# 

**Published:** 1847-10

**Authors:** 


PUBLISHED QUARTERLY.

PUBLISHED UNDER THE AUSPICES OF THE

**American Society of Dental Surgeons.**

EDITORS:

CHAPIN A. HARRIS, M. D., D. D. S.

AMOS WESTCOTT, M. D., D. D. S.

WILLIAM H. DWINELLE, D. D. S.

CORRESPONDENT FOR ENGLAND:

JAMES ROBINSON, D. D. S., 7 Gower-st. Bedford Square, London.

BALTIMORE:
ARMSTRONG & BERRY,
PHILADELPHIA: LINDSAY & BLAKISTON.
JOHN W. WOODS, PRINTER.

$5 00 per annum, payable in advance.

This Periodical weighs 9 ounces.

